# Using the Mutation-Selection Framework to Characterize Selection on Protein Sequences

**DOI:** 10.3390/genes9080409

**Published:** 2018-08-13

**Authors:** Ashley I. Teufel, Andrew M. Ritchie, Claus O. Wilke, David A. Liberles

**Affiliations:** 1 Department of Integrative Biology, Institute for Cellular and Molecular Biology, and Center for Computational Biology and Bioinformatics, The University of Texas at Austin, Austin, TX 78712, USA; wilke@austin.utexas.edu; 2 Department of Biology and Center for Computational Genetics and Genomics, Temple University, Philadelphia, PA 19122, USA; tuk03974@temple.edu (A.M.R.); daliberles@temple.edu (D.A.L.)

**Keywords:** evolutionary modeling, protein evolution, mutation-selection models

## Abstract

When mutational pressure is weak, the generative process of protein evolution involves explicit probabilities of mutations of different types coupled to their conditional probabilities of fixation dependent on selection. Establishing this mechanistic modeling framework for the detection of selection has been a goal in the field of molecular evolution. Building on a mathematical framework proposed more than a decade ago, numerous methods have been introduced in an attempt to detect and measure selection on protein sequences. In this review, we discuss the structure of the original model, subsequent advances, and the series of assumptions that these models operate under.

## 1. Introduction

Alignments of protein sequences and their mapping onto a phylogenetic tree show the role of mutational and selective pressures in generating variation across taxa. Accurately estimating the evolutionary distances between sequences as well as mechanistic parameters associated with distance estimation is crucial to understanding evolutionary processes [1,2,3,4]. However, estimating these distances with parameterized models necessitates the non-trivial task of constructing a mapping from genotype to phenotype [5]. The value of methods to bridge from genotype to phenotype has long been appreciated, and inspired the development of early codon-based models of protein evolution that work at the DNA level [6,7]. These models recognized the importance of considering non-synonymous and synonymous evolutionary rates separately, because a non-synonymous mutation alters a protein’s sequence and may have an effect on phenotype. The birth of mutation-selection models stems from applying these types of genotype to phenotype ideas to quantifying mutation-selection equilibrium [8].

## 2. Basic Structure of Model

Mutation-selection models rely on a central idea that changes in a DNA sequence can be thought of as the product of the probability of a mutation from one codon to another, the probability that the mutation fixes in the population, and an arbitrary scaling constant; such a model was first introduced by Halpern and Bruno [1] and parameterized directly from codon frequencies. For each codon in a sequence this allows for the definition of a 61 × 61 matrix of substitution rates. Thus,

(1) $$\begin{array}{c} \begin{array}{cc} r_{ab}^{i} & =k\times p_{ab}\times P_{\mathrm{fix}}^{i}\left(a,b\right),\;b\neq a,\\ r_{aa}^{i} & =-\sum_{b,b\neq a}r_{ab}^{i}, \end{array} \end{array}$$

where *k* is a scaling constant, $P_{\mathrm{fix}}^{i}$ is the probability that the mutation fixes and $p_{ab}$ is the probability that codon *a* mutates to codon *b*. The probability of a mutation between two codons can be expressed as

(2) $$\begin{array}{c} p_{ab}=\prod_{j=1}^{3}p_{a_{j}b_{j}}, \end{array}$$

where $p_{a_{j}b_{j}}$ is the product of the mutational probability of each nucleotide position *j* within a codon. Notably, the model can be streamlined by setting $p_{ab}$ equal to zero for double or triple mutations. To approximate the probability of fixation a weak mutation model [9] where mutations fix before the next one is introduced is used. This allows for the probability of fixation to be expressed as

(3) $$\begin{array}{c} P_{\mathrm{fix}}^{i}\left(a,b\right)=\frac{\ln(\frac{\pi_{b}p_{ba}}{\pi_{a}p_{ab}})}{1-\frac{\pi_{a}p_{ab}}{\pi_{b}p_{ba}}}, \end{array}$$

where $\pi_{a}$ and $\pi_{b}$ are the equilibrium frequencies. This expression explicitly relies on detailed balance assumptions. Using this probability of fixation permits for the calculation of position-specific substitution rates ($r^{i}$) from the mutation rates and the position-specific equilibrium frequencies of each codon. It should be noted that in the original model this is done without parameters. The equilibrium frequency of a codon can be calculated from the frequency of the amino acid that it codes for and the nucleotide frequencies, and these values were estimated from empirical data.

Other variations of this framework [10] have also been proposed using different formulations of the probability of fixation based on a diffusion approximation to the evolution of allele frequencies under a Wright-Fisher process. The probability of fixation for a haploid population was originally given as [11]

(4) $$\begin{array}{c} P_{fix}^{i}\left(a,b\right)=\frac{1-e^{-2s_{ab}}}{1-e^{-2Ns_{ab}}}. \end{array}$$

In this formulation, *N* is the population size and selective advantage is represented by the selection coefficient $s_{ab}$. When $|s_{ab}|$ is small the equation can be reduced as

(5) $$\begin{array}{c} P_{fix^{YN}}^{i}\left(a,b\right)=\frac{2s_{ab}}{1-e^{-2Ns_{ab}}}. \end{array}$$

The selection coefficient is here in linear space, while some formulas use a log-transformed coefficient.

Other expressions for the fixation probability are also possible. For example the Sella-Hirsh approximation [12],

(6) $$\begin{array}{c} P_{fix^{SH}}^{i}\left(a,b\right)=\frac{1-{\left(\frac{f_{a}}{f_{b}}\right)}^{2}}{1-{\left(\frac{f_{a}}{f_{b}}\right)}^{2N}}, \end{array}$$

where $f_{b}$ is the fitness of a single mutant allele against a wild-type population with fitness $f_{a}$.

Each of the above expressions relies on the assumption that mutation is weak and selection strong. More complex dynamics arise if these assumptions are relaxed and more sophisticated methods are required for the calculation of fixation probabilities under these conditions [13,14].

Using this modeling framework, distances between sequences can be calculated by multiplying $r^{i}$ by *t* and exponentiating the matrix. This allows for the use of maximum likelihood methods to estimate *t*, the evolutionary distance between sequences. While this framework was initially conceived to measure these evolutionary distances [1] the key idea that rate of codon change is a product of the mutation rate and the mutation fixation probability described in Equation (1) can also be used to detect and characterize selection in equilibrium and non-equilibrium frameworks.

## 3. Subsequent Implementations and Advances

While the Halpern and Bruno model [1] results in a set of site-specific matrices, this leads to an invalid assumption that sites behave independently. To help alleviate the reliance on the assumption of site independence, a technique to capture aspects of protein structure by employing a sequence-structure compatibility system [15] was introduced [16]. This model makes use of an empirical energy function that allows for non-synonymous substitution rates to be a function of how compatible a substitution is with a given structure. Additionally, this model was able to estimate selection to maintain sequence-structure compatibility with Markov chain Monte Carlo (MCMC) sampling. However, this model only considered pairs of coding nucleotide sequences, and was later expanded from considering only two taxa to *n* taxa [17]. Further, this expanded model incorporates information available from empirical amino acid replacement matrices and proposes a formulation at the amino acid level [17]. While these models use a single parameter to distinguish transitions and transversions, a further modification of the model moved to consider nucleotide exchangeabilities [18]. Other methods of capturing protein structure have also been applied. The use of a structurally constrained mean-field substitution model, which considers both unfolding and misfolding stability [19,20] was found to improve model fit over the model proposed at the amino acid level [21]. The speed of MCMC methods that augment substitution histories and assume site-independence was substantially improved by employing a partial sampling technique [22]. Additionally, it is also possible to map between selective coefficients estimated by mutation-selection frameworks and *dN/dS* values [23].

A further expansion of this modeling framework moved to consider site-specific fitness parameters as random effects by employing a Dirichlet Process (DP) Bayesian framework known as MG-MutSelDP [24]. This implementation of a site-specific mutation-selection model is widely used and currently available through the software package PhyloBayes [25]. A competing method, known as the swMutSel model, was introduced shortly after the MG-MutSelDP model. It employs a maximum penalized-likelihood (MPL) technique [26] and uses a one-model-per-datum approach to describe site-specific fitness. This approach results in a highly parameterized model. For a protein-coding gene of length L codons, it estimates 19 × L site-specific fitnesses. The distinguishing difference between MG-MutSelDP and swMutSel is in how they consider site-specific fitness. The swMutSel model assumes that the site-specific fitness parameters are different at each site. The MG-MutSelDP model describes site-specific fitness parameters as random effects and assumes there is a set of site categories and each specific site is considered to have been generated by one component of a mixture of these categories. To the extent that there are general modes by which evolution interacts with biophysics to describe amino acid substitution, in principle random effects models should be able to identify these discrete modes of action. However, on the other side, the biophysics can be dependent on precise geometric details of interaction (such as interaction distances and side chain angles), so that in practice, this becomes a continuous space of context-dependent modes of interaction [27].

Which of these two models performs better has been a point of contention. The swMutSel model has been criticized for over-parameterization, while the MG-MutSelDP model avoids over-fitting and statistical inconsistencies associated with highly parameterized models [28]. In response the swMutSel model was then updated to use likelihood penalizing functions to partially address this issue [29]. Ultimately, the result of choosing to describe site-specific fitness in the highly parameterized way via the swMutSel model leads to estimates of a large proportion of highly deleterious scaled selection coefficients. It has been argued that these estimates are an erroneous artifact of model overparameterization [28]. The random-effects framework of the MG-MutSelDP model estimates that all scaled selection coefficients are either nearly neutral or weakly deleterious [30]. However, the MG-MutSelDP framework can produce misleading results for certain sites due to inappropriate prior distributions resulting in cases where the amino acid predicted to be the most highly abundant is in fact not abundant at all [28]. Despite the over-parameterization of the swMutSel model, this model is found to give slightly more reliably estimated distributions of selection coefficients, though both methods perform similarly [30]. Notably, this comparison was done using simulations of 512 taxa on a balanced tree with equal edge lengths and the relative performance of these models depends on the underlying data. In information-poor settings, the over-parameterization of the swMutSel model may be more problematic. Considering that both methods for estimating site-specific selective coefficients from alignments suffer from pitfalls, a method to experimentally measure site-specific selection coefficients was introduced [31] and this method has been shown to significantly improve modeling in specific cases [32,33].

The descriptions of proteins present a characterization of protein evolution based on site-wise descriptions of amino acids. These are treated as site-independent and in equilibrium. Equilibrium assumptions characterize fixed evolutionary constraint and the consequences of this assumption are important. Similarly, while relaxing site-specific constancy of selective pressure is meant to accommodate compensatory processes associated with non-independence, this treatment ultimately breaks down and will also be discussed.

## 4. Equilibrium Assumptions and Likelihood

The original Halpern and Burno mutation-selection model [1] was formulated under the simplifying assumption that amino acid fitnesses at each site would remain constant over time and across the phylogeny, giving rise to a stationary process of evolution. Biologically, this implies a situation in which genes begin near their optimal state, balanced by occasional mildly deleterious mutations that tend to be removed over time, and do not undergo significant adaptation or compensatory covariation over the timescale of interest. It was further assumed that in this equilibrium state, the mutation-selection process was time-reversible. These assumptions allowed the formulation of fixation probabilities in terms of simpler and more biologically meaningful parameters through the relation $\frac{P_{\mathrm{fix}}\left(a,b\right)}{P_{\mathrm{fix}}\left(b,a\right)}=\frac{\left(\pi_{b}p_{b}a\right)}{\left(\pi_{a}p_{a}b\right)}$, and permitted efficient estimation of phylogenetic relationships via maximum likelihood.

Assuming that sequences were at equilibrium suited the original purpose of the models in reconstructing evolutionary distances from coding sequences. However, the assumptions are unrealistic, not only for genes that are likely to have been affected by directional selection, but also as a description of genes subject to compensatory processes (i.e., all protein-encoding genes where the folded structure is important). This renders the original models unsuitable for detecting adaptation or inferring site-wise changes in fitness. Nevertheless, the explicit modeling of the relationship between relative fitnesses and evolutionary rates makes an attractive foundation from which to address this problem, as it has roots in an underlying population genetic process. Applications aimed at inferring fitnesses from mutation-selection models build on older methods for detecting adaptation through estimating the mean ratio of the rates of non-synonymous and synonymous substitutions (*dN/dS*); site- and residue-specific fixation probabilities $P_{\mathrm{fix}}^{i}\left(a,b\right)$ more closely model the processes giving rise to this quantity [34].

Especially when using mutation-selection models to detect adaptation, we must relax the assumptions of stationarity and reversibility. This requirement has several consequences for methods development. In the first instance, the detailed balance relation between fixation probabilities and equilibrium frequencies given above no longer holds in general. This formulation may still be used, but the interpretation of the parameters is altered. Following an event that modifies the relative fitnesses of amino acids at a set of similar sites, the distribution of amino acids at those sites will no longer equal the distribution at equilibrium. The parameters $\pi_{a}$ no longer represent the distribution of observable quantities, but rather the stationary amino acid frequencies to which the new process will ultimately converge if not disturbed (the instantaneous equilibrium frequencies of the site). In practice, this means that an extra vector of parameters is required to represent amino acid frequencies at the root, since these cannot be identified with any parameters of the branch-specific models [35], assuming no part of the tree shares the selective process that generated the root sequence. If adaptive events are to be modeled along the tree, this will also require estimation of a new set of parameters following each event. Since the rates of transition between amino acid pairs are described in terms of these parameters, these will also change over the tree, rendering the overall process inhomogeneous over time.

How changes over time in amino acid fitness profiles are modeled requires consideration. Time-inhomogeneous models of nucleotide evolution have a deep history in phylogenetics. These models have been extensively investigated in attempts to account for observed differences in nucleotide composition in the genomes of related species. The initial proposal allowed the application of an arbitrary transition matrix to each branch of the tree [36]. This model suffers from the theoretical issue that it is not identifiable in the most general case; that is, no amount of sequence data will allow a unique estimate of the nucleotide frequencies or transition probabilities unless certain conditions are placed on the model [37].

The identifiability problem that has received the most attention is that the fully general model cannot discriminate among different labellings of the states at internal nodes [38]. This appears to be a DNA-specific problem. For DNA, if the frequencies of nucleotide A at each node are swapped for the frequencies of C, and the transition probabilities $P_{Aj}$ and $P_{Cj}$ are swapped for each destination nucleotide *j* on each branch of the tree, the new model will give the same joint distribution of nucleotides at the leaves. This scenario is extremely unlikely when fitting models designed for protein-coding sequences because the genetic code dictates transition probabilities among codons that are then affected by the equilibrium fitnesses and corresponding fitnesses, meaning that the rows of the transition matrix cannot be swapped. Aside from this, additional factors which may raise identifiability concerns include trees that have nodes of degree two (breakpoints in the middle of branches for example) and cases in which more than one instantaneous rate matrix can generate the same branch transition probabilities [37,39].

Assuming that these theoretical concerns can be addressed, the large number of parameters involved in nonhomogeneous models means that estimation can be difficult in practice. An early application attempted to account for compositional differences among related species by retaining a global relative rate matrix (for example, dictated by the genetic code) and only allowing base frequencies to vary among branches [40]. In principle, this method is well justified, as the mutational process is generally conserved and is distinct from the selective process that gives rise to different frequencies. However, it remains computationally demanding and prone to over-fitting [41]. Accordingly, subsequent methods made efforts to reduce the computational burden through using a single composition parameter representing GC content (or HP, hydrophobic and polar amino acids, in a protein context if needed) or otherwise reducing the parameter space [35,42] or by selecting from a smaller number of models that are shared across multiple branches of the tree [43,44].

**Figure 1:**
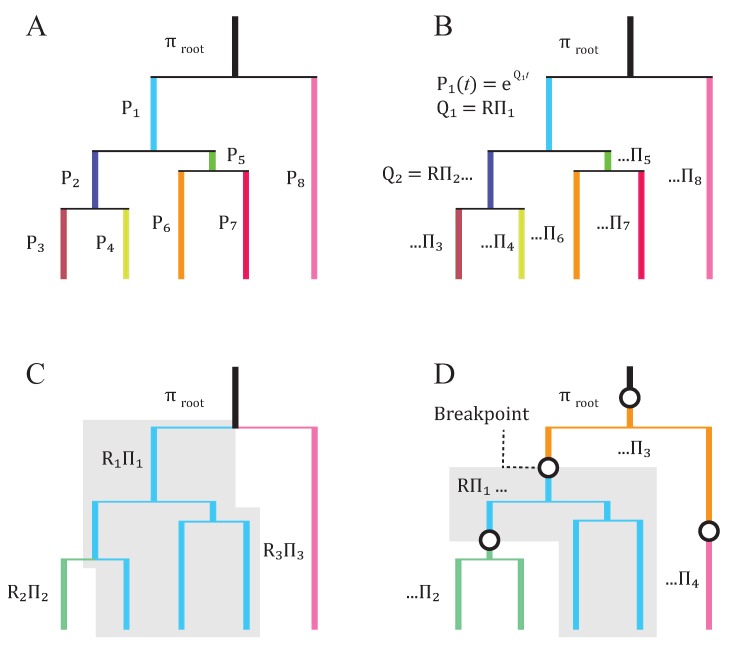
Illustration of the variety of nonstationary and non-time-homogeneous phylogenetic models. (**A**) The Barry-Hartigan model is the most general possible Markov substitution model. The substitution process is modeled by an arbitrary Markov transition matrix for each branch (P${}_{1}$–P${}_{8}$, indicated by branch colors) and a vector of initial frequencies at the root $\pi_{root}$. (**B**) A homogeneous but nonstationary model. Transition matrices are derived from a reversible continuous-time Markov model. All branches share the same rate matrix R, but the stationary frequencies $\Pi_{i}$ are permitted to vary across the tree. (**C**) A non-homogeneous model reflecting more recent methods whereby a small number of reversible Markov models with different rates and frequencies are assigned to multiple branches within the tree (shaded region). (**D**) A nonstationary ‘breakpoint’ model in which state frequencies may differ within as well as among lineages.

A major innovation was the development of the break point (BP) model [41]. This type of model allows shifts in selective pressures to occur at any point in the tree, not only at speciation, duplication, or lateral transfer event nodes [45]. Like their forebears, the breakpoint models are not designed to detect adaption or infer values related to fitness or selective pressure. They do not explicitly model fixation probabilities or variable transition rates, and effectively integrate over break point numbers and positions. However, they remain some of the most complete methods for modeling non-stationary evolution. While it may not be critical to change models over a branch, when averaging a process over a branch, an important consideration is if the model that is fit over the branch reflects the combination of processes and associated parameters accurately [46]. A comparison of each of these types of nonstationary non-time-homogenous models is given in Figure 1.

Another consequence of relaxing the stationarity assumption is that standard phylogenetic optimization methods make strong use of time reversibility to allow efficient computation of model likelihoods over a tree. Given a time-reversible model of sequence evolution, the root may be considered as being anywhere on the tree without altering the likelihood. Using the standard phylogenetic likelihood algorithm [47], partial likelihoods for each state at the head of each subtree can be calculated recursively and stored. During optimization, the tree likelihood is recalculated after an update that alters the branch lengths or the tree topology, and the root of the tree can be placed so that only a minimum number of partial likelihoods need to be recomputed.

**Figure 2:**
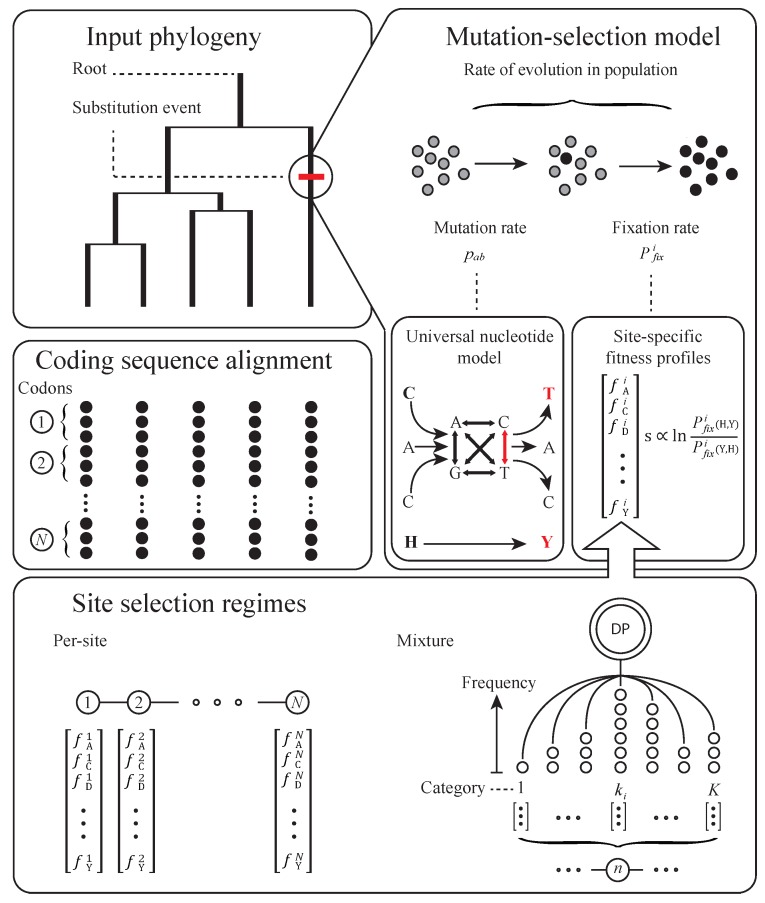
Schematic showing the construction of mutation-selection models in an equilibrium framework. Clockwise from top left: the model describes the DNA substitution process within a protein-coding sequence along a rooted phylogenetic tree. Each substitution is modeled by the product of a mutation rate and the rate of fixation within a population. The rate of mutation among codons is represented by the product of transition rates at each codon position. The fixation rate is represented by a population genetic model operating on a selection coefficient derived from a set of site-specific amino acid fitness values (AA values shown as single-letter codes). Fitness values may be treated as distinct parameters for each codon site, or probabilities may be calculated via a mixture over an assumed or unknown number of site categories (random effects). In a Bayesian framework the prior distribution of an unknown set of site categories may be given by a Dirichlet Process (DP).

Under a non-reversible model, the position of the root cannot be altered, meaning that recalculation after an update will require more computation [40]. Solutions to this problem require caching an additional upper partial likelihood for each branch in the tree, which minimizes recomputation after branch length optimization and topology updates through nearest-neighbor interchange [48]. If the model includes break points, these can be considered extra nodes with a single descendant subtree and the likelihood calculated as normal, allowing for the new amino acid fitness values (Figure 2). Update mechanisms that add, delete or move break points or change frequencies within a lineage will also require recomputation of likelihoods, sometimes over several nodes, although this is mitigated in BP models by ensuring that frequencies before and after a break point are independent. Models designed for reconstructing phylogenies typically include topology update mechanisms, including moves that the change the position of the root [41]; however, due to the highly parametric nature of time-variable mutation-selection models for amino acid data and the additional complexity created by non-stationarity, it is likely that practical methods in a maximum likelihood framework will need to rely on fixed topologies in the near future. This type of method is appropriate where traditional Markov substitution models can be trusted to estimate the proper topology with subsequent branch length optimization under the new model. Branch lengths estimated under a nonstationary model do not have the standard interpretation relating to the average substitutions per site but can be rescaled so as to give this interpretation [39,49].

## 5. Biochemical and Population Genetic Assumptions

While numerous advances in the complexity of mutation-selection models continue to be proposed, they all make a series of assumptions. The original Halpern and Bruno model [1] assumes that most positions are under purifying selection, implying that there are only a few amino acids have comparatively high fitness values. Further, it is assumed that this selective pressure is constant over time (reflecting diversifying rather than directional selection for positive *s* values). Therefore, these assumptions results in lack of consideration of site-interdependence. However, sites do not evolve in a vacuum and the fitness of sites are indeed interdependent for numerous reasons.

At the level of codons, codon usage can vary dramatically across a gene. Position-dependent codon usage bias has been observed in numerous taxa [50,51,52] and individual codon usage biases follow a position-dependent exponential decay model [53]. It has been theorized that codon that translated less efficiently are present in the first 90–150 nucleotides to create a “ramp” to slow elongation rates and prevent ribosomal traffic jams [54]. Whatever the underlying cause of site dependent codon usage, selection on synonymous codon usage may vary depending on the translational efficiency of nearby codons. Additionally, messenger RNA (mRNA) translation rates are coupled to proper protein folding and function [55]. As mentioned in the original paper [1], mutation-selection models with site-specific selection parameters are in principle capable of modeling these processes by decomposing fitness parameters into codon and amino acid components [26]. In practice, the framework has been adapted to study codon usage by applying fitness effects to synonymous as well as nonysynonymous changes [10,56], and these have been demonstrated to outperform simpler codon models on some data sets [57,58].

Accounting for protein structure leads to yet another level of site interdependence. In order for a protein to function it must be able to fold into a stable conformation. The stability of a folded protein (ΔG) depends on the difference in free energy between the native and unfolded forms of the protein. Further, proteins do not exist in isolation and their function often depends on the protein’s ability to bind other proteins specifically. The stability of binding can be quantified in a similar manner to that of protein stability, by measuring the difference in free energy between bound and unbound and non-specifically bound proteins. While these measures can be combined into a single fitness metric [27,59] they are still considered independently. Further, it is unclear how to appropriately weight fitness contributions of binding and stability. Another issue arises when considering these sorts of metrics as measures of fitness over long evolutionary time periods, as significantly divergent sequences are not guaranteed to fold in a specific set structure. Hence, the use of ΔG as a fitness metric has limited ability to quantify the relationship between site-interdependence and selection [4]. The environment around a protein offers a further level of selective pressure on sites, including the chemical make up the surrounding solvent, protein concentration, temperature, and the presence of off-target interacting partners [4,60,61].

Beyond these physical aspects that result in site interdependence, there is a temporal aspect as well. Epistatic interactions result in changes to a site’s fitness landscape as other interacting sites are modified. The fitness landscapes at these sites tend to change to stabilize the current state [62,63], a phenomenon referred to as entrenchment or an evolutionary Stokes shift. The constant shifting of site specific fitness landscapes imposed by interacting sites will tend to result in underestimations of *dN/dS* [34].

In addition to the biochemical constraints that result in site interdependence, there are also population level processes at play. Mutation-selection models have two components, a mutational probability and a fixation probability. The fixation probability can be boiled down to two key parameters, the effective population size ($N_{e}$) and the selection coefficient (*s*). The standard treatment from the model makes specific assumptions about the nature of both $N_{e}$ and *s*. Phylogenetic implementations of mutation-selection models treat substitutions between amino acids at a site as being constant over the length of a branch. In some cases, this will lead to an averaging effect of selective strengths. This time averaging effect is equally acute for effective population sizes as it is for selective coefficients. Further, there are multiple effective population size parameters that matter, as the introduction of new mutations behaves differently from the fixation of mutations and occurs on different time scales for the same proposed mutation [64]. Lastly, the modeling framework makes a strong assumption about weak mutational processes and the dynamic can be very different when mutational pressure is strong [14].

## 6. Conclusions

Though significant progress has been made towards using mutation-selection models to quantify selection on protein sequences, these models still suffer from a number of shortcomings. The basic framework of the model necessitates a set of biologically unrealistic assumptions. While many have introduced methods to account for certain aspects of violating these assumptions, further work towards constructing realistic models of evolution and using these models to quantify selection is necessary. However, constructing a modeling framework that accounts for a lack of evolutionary equilibrium as well as the biochemical and population level influences on protein evolution is a non-trivial task. Accounting for non-equilibrium processes is necessary for characterizing positive directional selection. Other assumptions about the nature of selection on protein structure and function, including compensatory amino acid substitution, and on the complexity of the underlying population genetics that are not being modeled await further modeling to determine the magnitude of incorrect inference. Lastly, branch averaging model mis-specification effects for heterogeneous processes appear both difficult to account for and to be small in affect, although averaging of negative and positive selection along a branch leads to reduced power (to detect positive selection) with increased branch length. Even with large data sets, complex models struggle with various parameter estimation problems and the ultimate solution may involve finding the right balance of biologically meaningful parameters and assumptions that is tractable [61,65].
